# NETosis and Neutrophil Extracellular Traps in COVID-19: Immunothrombosis and Beyond

**DOI:** 10.3389/fimmu.2022.838011

**Published:** 2022-03-02

**Authors:** Yuanfeng Zhu, Xiaoli Chen, Xin Liu

**Affiliations:** Clinical Medical Research Center, Southwest Hospital, Army Military Medical University, Chongqing, China

**Keywords:** NETosis, NETs, COVID-19, immunothrombosis, post COVID-19 syndrome, immunopathology

## Abstract

Infection with SARS-CoV-2, the causative agent of the Coronavirus disease 2019 (COVID-19) pandemic, causes respiratory problems and multifaceted organ dysfunction. A crucial mechanism of COVID-19 immunopathy is the recruitment and activation of neutrophils at the infection site, which also predicts disease severity and poor outcomes. The release of neutrophil extracellular traps (NETs), occurring during a regulated form of neutrophil cell death known as NETosis, is a key effector function that mediates harmful effects caused by neutrophils. Abundant NETosis and NET generation have been observed in the neutrophils of many COVID-19 patients, leading to unfavorable coagulopathy and immunothrombosis. Moreover, excessive NETosis and NET generation are now more widely recognized as mediators of additional pathophysiological abnormalities following SARS-CoV-2 infection. In this minireview, we introduce subtypes of NET-producing neutrophils (e.g., low-density granulocytes) and explain the biological importance of NETs and the protein cargos of NETs in COVID-19. In addition, we discuss the mechanisms by which SARS-CoV-2 causes NETosis by upregulating viral processes (e.g., viral entry and replication) as well as host pro-NET mechanisms (e.g., proinflammatory mediator release, platelet activation, and autoantibody production). Furthermore, we provide an update of the main findings of NETosis and NETs in immunothrombosis and other COVID-19-related disorders, such as aberrant immunity, neurological disorders, and post COVID-19 syndromes including lung fibrosis, neurological disorder, tumor progression, and deteriorated chronic illness. Finally, we address potential prospective COVID-19 treatment strategies that target dysregulated NETosis and NET formation *via* inhibition of NETosis and promotion of NET degradation, respectively.

## Introduction

Coronavirus disease 2019 (COVID-19) is a global pandemic caused by severe acute respiratory syndrome coronavirus-2 (SARS-CoV-2) infection (1). Pneumonia is a typical symptom of COVID-19 infection, while acute respiratory distress syndrome (ARDS) and multiple organ failure are common in severe COVID-19 patients (2). Immunopathological manifestations, including cytokine storms and impaired adaptive immunity, are the primary drivers behind COVID-19, with neutrophil infiltration being suggested as a significant cause. It has been observed that neutrophil extravasation occurs widely in pulmonary capillaries, myocardia, and the liver in post-mortem examinations of COVID-19 patients (3). Furthermore, SARS-CoV-2 infection has also been linked to increased neutrophil-to-lymphocyte ratios, which is associated with disease severity and clinical prognosis (4, 5). As a result, neutrophils and their effector mechanisms (e.g., degranulation, oxidative burst, and NETosis) are increasingly recognized as important mediators in the immunopathogenesis of COVID-19 (6).

NETosis is a special form of programmed cell death in neutrophils, which is characterized by the extrusion of DNA, histones, and antimicrobial proteins in a web-like structure known as neutrophil extracellular traps (NETs) (7). NETosis is induced by a range of microbial stimuli and proinflammatory mediators [e.g., interleukin (IL)-8 and IL-1β] (7). The increased generation of reactive oxygen species (ROS) is a crucial intracellular process that causes NETosis (8). ROS trigger proteases including protein arginine deiminase 4 (PAD 4), neutrophil elastase (NE), and Gasdermin D that catalyze the process of chromatin decondensation, nucleus disintegration, and cell rupture (7). Although NETs are important for preventing pathogen invasion, their excessive formation can result in a slew of negative consequences, such as autoimmune inflammation and tissue damage (9). When NETs are activated in the circulation, they can also induce hypercoagulability and thrombosis (10).

Previous studies have shown that NETosis and NET release are both elevated by circulatory or infiltrating neutrophils in COVID-19 patients (11, 12). Meanwhile, it has also been demonstrated that SARS-CoV-2 infection can directly induce NETosis and NET release in healthy neutrophils (13). Furthermore, NET production is regarded as a predictor of disease severity (11, 14) and clinical outcomes (15) in COVID-19. A key mechanism of NETosis and NET-related disease is the induction of immunothrombosis (16). However, NETosis and NETs are also increasingly recognized as mediators of other pathophysiological changes, such as immune dysfunction, neurological abnormities, and post COVID-19 disorders. Herein, we attempt to describe the biological significance of neutrophils and NETs in COVID-19 and the mechanisms involved in SARS-CoV-2-induced NETosis and NET release. We also discuss the roles of NETosis and NETs in immunothrombosis and other COVID-19-related disorders. In addition, we address future potential COVID-19 therapeutic options that target dysregulated NETosis and NET formation.

## The Biology of Neutrophils and NETs in COVID-19

Maturation is an important aspect of neutrophil biology that significantly impacts NETosis and NET formation (17, 18). Mature neutrophils generally have a greater capacity to produce NETs in response to external stimuli (19, 20). However, a group of heterogeneous population of both mature and immature neutrophils, known as low-density granulocytes (LDGs), has recently been shown to be more prone to spontaneous NETosis and NET production in autoimmune disorders (21, 22). Importantly, LDG ratios are increased in individuals with COVID-19. The amounts of LDGs are further upregulated in individuals with severe COVID-19 where it is indicative for poor clinical prognoses (23–25). Furthermore, LDGs are prone to NETosis and NET formation, thereby contributing to COVID-19-related immunothrombosis and organ injury (26). Additional research has shown that a subset of immature (low CD10 expression, CD10^low^) LDGs display reduced capacity to produce NET and robust immunosuppressive capabilities that resemble myeloid-derived suppressor cells (27). In contrast, mature LDGs with higher CD10 and CD16 expression seem to be preferentially enhanced in COVID-19 and are more prone to NETosis and NET production (24, 27). For example, a unique LDG subset with intermediate expression of CD16 (CD16^int^) was clearly observed in COVID-19 patients. These LDGs spontaneously produce NETs and are favorably associated to lung inflammation and overall disease severity (23). Another study demonstrated the relationship between a cluster of CD33^low^CD16^+^CD11b^+^ LDGs and increased detection of myeloperoxidase (MPO)-DNA, suggesting that this population plays a direct role in NETosis in COVID-19 (24). In addition, the presence of CD33^low^CD16^+^CD11b^+^ LDGs is also associated with disease severity and prognosis.

NETs carry a wide range of protein cargos, which are either directly derived from the nucleus and granules or captured during the process of NET formation (28). These protein cargos are necessary for enabling NETs to exert their microbicidal effector function and cause pathological injury. In COVID-19, major NET protein cargos of NETs (i.e., NE, MPO, and histones) are significantly elevated. These protein cargos are associated with increased expression of proinflammatory mediators [e.g., IL-6, IL-8, and C-X-C motif receptor 2 (CXCR2)], multiple organ damage, increased risk of ventilation, and short-term mortality (29, 30). Other prothrombotic factors or proinflammatory damage-associated molecular pattern (DAMP) cargos are also enriched in NETs (27, 31). Intriguingly, Skendros et al. showed that COVID-19 neutrophils produce NETs carrying tissue factor (TF) (32). Moreover, platelet-rich plasma isolated from COVID-19 patients also stimulates the production of TF-containing NETs from healthy neutrophils, leading to thrombotic activity in human aortic endothelial cells *in vitro* (32). A separate study showed that the expression of high mobility group box 1 (HMGB1)–DNA complexes is upregulated in NETs released by COVID-19 patients, suggesting that circulating NETs contain higher levels of DAMPs (27). Taken together, these findings clearly illustrate the involvement of prothrombotic and proinflammatory cargos in NET-induced COVID-19 pathology.

## Mechanisms of SARS-CoV-2-Induced NETosis and NET Formation

The establishment of intracellular infection is necessary for SARS-CoV-2 to directly induce NETosis and NET release in neutrophils. The first prerequisite is the successful neutrophil infiltration by the virus. Neutrophils with higher viral antigen loads promote more efficient NET production, while this process is hindered by inhibition of the classical entry mechanism involving angiotensin converting enzyme 2 (ACE2) and serine proteases (13). However, SARS-CoV-2 can also infect host cells through noncanonical receptors such as C-type lectin receptors (33, 34), which have recently been shown to mediate pattern recognition of dengue virus and NET formation (35). This suggests that C-type lectin receptors may also be involved in mediating NET release in COVID-19. In addition, NETosis and NET formation are also influenced by the viability and intracellular replication of SARS-CoV-2. When infected with viable rather than inactivated viruses, neutrophils are considerably more likely to undergo NETosis and produce NETs (13, 36). Furthermore, targeted pharmacological inhibition of viral replication reduces NET formation (13). Another mechanism behind SARS-COV-2-induced NETosis and NETs lies in the fact that SARS-CoV-2 infection causes neutrophils to increase the production of pro-NETosis mediators. Indeed, SARS-CoV-2 can increase oxidative burst in neutrophils and inhibit the antioxidant response, thereby aggravating immunopathologies in COVID-19, including NETosis and NETs (8, 37). For example, Arcanjo et al. confirmed that SARS-CoV-2 triggers NETosis in human neutrophils through increased ROS production (36). In another recent study, a self-sustained autocrine production loop of IL-8, another intrinsic and essential driver of NETosis, was discovered in pulmonary and peripheral blood neutrophils, which promotes NET formation and indicate the severity of COVID-19 (38).

SARS-CoV-2 is significantly more effective at inducing NETosis *in vivo* than in cultured neutrophils (35). Furthermore, soluble substances in the plasma of COVID-19 patients also mediate NET formation in neutrophils from healthy people (39). These findings implicate indirect mechanism(s) underlying NETosis and NET formation induced by SARS-CoV-2. In fact, SARS-CoV-2 may stimulate the production of proinflammatory mediators or induce the release of DAMPs when contacting epithelial cells and other neighboring cells (e.g., macrophages) in the airway, resulting in a massive amplification of inflammatory and chemotactic responses. Proinflammatory mediators such as IL-8 and IL-1β are important NET-inducing mediators, and they are produced abundantly by SARS-CoV-2-infected epithelial cells and macrophages, increasing NETosis in tissues and intravascular neutrophils (40, 41). Another indirect route of SARS-CoV-2-induced NET production is platelet activation, which can enhance this process by interacting with neutrophils through toll-like receptor 4 (TLR4), platelet factor 4 (PF4), and extracellular vesicle-dependent processes (35, 42, 43). SARS-CoV-2 and its components (e.g., spike proteins and viral RNA) attach to platelets and increase their activation and aggregation in COVID-19, resulting in vascular injury and thrombosis, both of which are linked to NET formation (44, 45). For example, Wu et al. discovered NETs and overwhelming thromboses in the pulmonary tissues of individuals who had died from COVID-19 (43). The authors also discovered low viral load in the biopsied lungs from these individuals, as well as elevated PF4 expression, leading them to hypothesize that NET formation may be caused by activated platelets rather than SARS-CoV-2 itself. Anti-SARS-CoV-2 antibody or autoantibody overexpression is another typical indirect mechanism driving NETosis and NET formation. IgA2 antibodies were shown to be particularly high in severe SARS-CoV-2 infection, which correlates with circulating extracellular DNA. This, according to Staats et al., is indicative of NET development and predictive of catastrophic outcomes in COVID-19 patients (46). Other studies showed that autoantibodies against phospholipids and phospholipid-binding proteins (aPL antibodies) or PF4 are raised by SARS-CoV-2 infection (47) or vaccination (48). These autoantibodies then trigger NET release in neutrophils isolated from healthy individuals. COVID-19 also leads to the production of anti-NET autoantibodies, which are linked to increased circulating NETs and thromboinflammation in patients. Notably, autoantibodies have been shown to prevent NETs from being degraded by healthy control serum, allowing them to persist in individuals with COVID-19 (49).

## NETosis and NETs in Coagulopathy and Immunothrombosis in COVID-19

Immune cells like neutrophils interact directly with platelets and plasma coagulation factors, causing coagulopathy and thrombosis in COVID-19 patients, a condition known as immunothrombosis (39, 50). In COVID-19 patients, abnormal coagulation is common, and in most cases, disseminated intravascular coagulation (DIC) was observed in individuals with COVID-19 during hospitalization before they eventually died (51). Pathological findings also indicated that neutrophil-produced NETs are major elements of micro- and macro-vascular thrombi (52, 53). In addition, the collaboration of numerous components in NETs, including platelets, endothelial cells, coagulation factors, and inorganic polyphosphate, is required for NET-induced intravascular coagulation (32, 54). In COVID-19 and ex-COVID-19 cases, the major components of NETs, such as genomic DNA and citrullinated histone H3, have been suggested as coagulation inducers. For example, when cell free-DNA from human neutrophils spikes into plasma, it triggers thrombin generation by binding to factor XII (FXII), which is enhanced by diminished anticoagulant factors and poor fibrinolysis (55, 56). Complements also stimulate tissue factor production and interact with the platelet/NETs/thrombin axis, making them important players in COVID-19 immunothrombosis (32, 57). Furthermore, histones boost thrombin synthesis in a dose-dependent manner, in a process that relies on activatable platelets as well as platelet activation indirectly, but not by platelet tissue factor (58). Other components, such as neutrophil elastase and cathepsin G *via* protease-activated receptors, and neutrophil elastase can also degrade tissue factor pathway inhibitors to promote coagulation (59). NETs and histones may also stimulate platelets to be procoagulant by upregulating the expression of P-selectin (54).

Within COVID-19 patients, NETs can cause microvascular thrombosis, tissue damage, and organ failure (60, 61). NETs are released into the intravascular circulation, bind to vessel walls, capture platelets and microvesicles, and subsequently obstruct blood flow. Endothelial *in situ* microvascular thrombosis, which may be induced by NETs, is one cause of thrombosis in COVID-19 (62). Type I interferons (63) and the NOD-like receptor protein 3 (NLRP3) inflammasome (64) are two examples of proinflammatory pathways that can be triggered by NET formation. In addition, the cytotoxic action of the enzymes and histones that are released from NETs can promote endothelial cell death and endothelial dysfunction (65, 66), which is considered a critical pathogenic mechanism of organ injury in COVID-19 patients (67, 68). Moreover, NET production promotes lung epithelial cell death and intravascular thrombus formation, thereby crucial for inducing acute lung injury in COVID-19 (69, 70). Indeed, due to the presence of abundant pulmonary vascular neutrophils and dynamically released platelets, NET-induced immunothrombosis is more likely to occur, resulting in occlusion of the microvasculature and ischemic injury (16, 71). Autopsies of people who have died from COVID-19 have also revealed occlusive thrombi inside the pulmonary vasculature (72). These autopsies have also shown that incidences of alveolar capillary microthrombi in COVID-19 patients are significantly higher than in influenza patients (72). NETs may also contribute to renal failure and liver injury in COVID-19 patients due to their pro-thrombotic activity. Numerous microthrombi in the hepatic sinusoids, as well as ischemic-type hepatic necrosis, are found in the postmortem livers of COVID-19 patients (73, 74). Furthermore, inflammatory microvascular thrombi, which contain NET components and platelets, have been observed in the lungs, kidneys, and hearts of COVID-19 patients (75).

## NETosis and NETs in the Pathology of COVID-19 Beyond Immunothrombosis

NETosis and NETs are increasingly recognized as causes of vascular injury that induce immunothrombosis, as summarized in the preceding section. In addition, autoantigens, proinflammatory mediators, proteases, and cytotoxic enzymes are exposed during NETosis, leading to aberrant immunity such as cytokine storms, autoimmune disorders, and immunosuppression. NETosis and NETs may also have a role in the development of post COVID-19 syndromes, including lung fibrosis, neurological disorders, tumor growth, and worsening of concomitant diseases.

### Cytokine Storm

In sepsis (76) and sterile inflammatory conditions (28, 77), NETs and other by-products of NETosis have been shown to act as direct inflammation amplifiers. Hyperinflammation (also known as a “cytokine storm”) is typical in COVID-19 and works in tandem with immunothrombosis to promote ARDS and extensive organ failure (78, 79). According to Ouwendijk et al., NET-specific MPO–DNA complexes in patient plasma corelate with SARS-CoV-2 viral load and plasma inflammatory markers [e.g., C-reactive protein (CRP) and IL-6], implying a NET-associated cytokine storm in COVID-19 (80). In a more recent study, Torres-Ruiz and colleagues directly simulated monocyte-derived macrophages with NETs and NET protein cargos extracted from COVID-19 patients, revealing a significant increase in major proinflammatory mediators such as IL-6, IL-8, IL-17A, tumor necrosis factor α (TNF-α), and granulocyte macrophage colony-stimulating factor (GM-CSF) (27). NETs are also thought to trigger IL-1β secretion in monocytes/macrophages, resulting in a signaling loop that augments the resolution of inflammation after SARS-CoV-2 infection (41). Furthermore, SARS-CoV-2 drives NETosis and NET formation to allow for the release of free DNA and by-products (e.g., elastases and histones). This may trigger surrounding macrophages and endothelial cells to secrete excessive proinflammatory cytokines and chemokines, which, in turn, enhance NET formation and form a positive feedback of cytokine storms in COVID-19 (81, 82).

### Autoimmune Diseases

NET release enables self-antigen exposure and autoantibody production, thereby increasing the autoinflammatory response (83). In COVID-19, SARS-CoV-2 infection may also induce autoimmune symptoms through mechanisms involving NET production (83, 84). First, SARS-CoV-2-induced NET production may be a key source of autoantigens. Virus-affected protein autoantigens have also been used to study autoimmune sequelae in COVID-19 in a series of multi-omic investigations (85–87). The authors in these studies proposed a pathway for virus-induced protein modifications that generate autoantigens, thereby suggesting NET-associated protein alterations (e.g., citrullinated histones) as autoantigens in COVID-19 (88). Furthermore, Wang et al. reported the discovery of unknown autoantigens related to nucleic acid and nucleocytoplasmic transport in COVID-19. As NETs carry substantial amounts of nuclear components, their role in this process is plausible (87). Second, anti-NET antibodies and other autoantibodies are elevated in COVID-19. For example, Zuo et al. tested anti-NET antibodies in 328 hospitalized patients with COVID-19 and found anti-NET IgG and IgM levels to be higher in patients than in healthy controls, which is also linked to poor clinical outcomes (49). In addition, Torres-Ruiz et al. found that patients with COVID-19 who have higher anti-NET antibodies are more likely to be detected with positive autoantibodies [e.g., antinuclear antibodies (ANA) and anti-neutrophil cytoplasmic antibodies (ANCA)], suggesting that COVID-19 NETs may act as potential inducers for autoimmune responses (27).

### Immunosuppression

COVID-19 patients have weakened adaptive immunity as well as a high level of inflammation (89). Although there is no clear evidence for NET-associated immunosuppression in COVID-19, it is worth noting that tumor-associated NETosis and NETs promote an immunosuppressive environment in which anti-tumor immunity is compromised (90, 91). NETs have also been shown to enhance macrophage pyroptosis in sepsis (92) and to be associated with endothelial apoptosis in COVID-19 patients (93), thereby further facilitating an immunosuppressive microenvironment. Furthermore, persistent immunosuppression may result in bacterial co-infection or secondary infection, which has emerged as a new threat for COVID-19 patients (94). For example, Kreitmann et al. found that early bacterial coinfections were more prevalent in COVID-19 patients than those infected with other viruses (95). In another study, de Buhr et al. presume that NETs and NET-degraded fragments may cause bacterial coinfections in COVID-19 patients (96). The authors’ hypothesis is based on their previous ex-COVID-19 studies, which showed that host intrinsic nucleases that degrade NETs and cell-free DNA may facilitate infection with certain bacteria (e.g., *Actinobacillus pleuropneumoniae*) that do not synthesize intrinsic nicotinamide adenine dinucleotides (NAD) *de novo* but instead rely on degraded NET products to obtain extrinsic NAD (97). In another ex-COVID-19 study, NETs in the cerebrospinal fluid of patients with pneumococcal meningitis were demonstrated to interfere with bacterial clearance (98). As a result, these findings may provide novel implications for the possibility of NET-induced immunosuppression in COVID-19 in the context of co-existing bacterial infection.

### Post-COVID-19 Syndrome

Patients infected by SARS-CoV-2 have also shown chronic sequelae for weeks or even months, a condition termed as “post COVID-19 syndrome” or “long COVID” (99, 100). Following initial onset of COVID-19, an estimated 50% or more of COVID-19 survivors may develop multi-organ problems (e.g., pulmonary dysfunction and neurologic impairment) or have worsening concomitant chronic illness (100–102). Notably, a recent study found that circulating markers of NETs returned to comparable levels with healthy controls after 4 months of infection, implying that NETs are persistent and may be involved in post COVID-19 syndrome pathology (30, 103, 104).

Pulmonary fibrosis is a common post COVID-19 respiratory conditions due to epithelial–mesenchymal transition (EMT) after SARS-CoV-2 infection and inflammation (105). Pandolfi et al. showed that NETs in the bronchoalveolar lavage fluid of severe COVID-19 patients cause EMT in lung epithelial cells (106). The authors also found that SARS-CoV-2 infection increases NETosis in co-cultured lung epithelial cells, macrophages, and neutrophils, as evidenced by increased SMA (a mesenchymal marker) expression and decreased E-cadherin (an epithelial marker) expression. Other studies reported that NETs and NET-associated proteins favor the expression of inflammatory and fibrotic genes (107), or increase the number and activity of fibroblasts (108) in ex-COVID-19 fibrosis induced by chemical or infectious stimuli. These data may indirectly implicate NETs as important players in the pathogenesis of pulmonary fibrosis post COVID-19.

Another common post-COVID-19 condition is persistent neurologic impairment (109, 110), which is characterized by cerebrovascular abnormalities and symptoms such as fatigue, myalgia, and cognitive impairment (111). Direct SARS-CoV-2 infection, persistent neuro-inflammation by central or peripheral mediators, and cerebrovascular abnormalities, all of which are linked to the excessive production of NETs, are among the mechanisms responsible for neurologic impairment following COVID-19 (102, 110). For example, Lou et al. highlighted the impact of SARS-CoV-2 infection on cerebrovascular disorders, citing increased NET formation and related immunothrombosis, inflammation, and antiphospholipid antibody production as major impacts (112). In addition, Pramitasuri et al. found NET-induced vasculopathy (e.g., procoagulant activity and endothelial dysfunction) and neuroinflammation (e.g., extruded NET components mediated neuronal damage, NETs-IL-1 loop, and IL-17 cascades) in ischemic stroke following COVID-19 (113). These findings could point to a potential link between NETs and COVID-19-related long-term neurological diseases.

COVID-19 also has a long-term influence on tumor progression (114). Patients with tumors have been shown to be more vulnerable to SARS-CoV-2 infection and subsequent development of severe COVID-19 (115). In addition, Saini et al. suggested that patients who have recovered from COVID-19 may have an increased risk of developing cancer or of cancer progression and metastasis (116). Moreover, NETs have been shown to change the tumor microenvironment (117), awaken cancer cells (118), and enhance tumor progression and metastasis (119). As a result, the persistent presence of NETs may mediate the long-term effects of tumor induction or worsening in COVID-19. This hypothesis, however, will need to be proven in future studies using more direct approaches.

COVID-19 may aggravate pre-existing chronic illnesses such as hypertension (120), obesity (121), and diabetes (122), in addition to having a direct effect on the host. Given the circulatory and infiltrating nature of neutrophils, the broad occurrence of NETosis and NETs in COVID-19 may facilitate the worsening of various chronic disorders. For example, Thierry et al. found that NETs and their by-products (e.g., elastase) may induce hypertension, thrombosis, and vasculitis in COVID-19 (82). Furthermore, hypertensive patients infected by SARS-CoV-2 have greater neutrophil counts than COVID-19 patients without hypertension, implying a potential relationship between NETs and COVID-19-related hypertension (120). Moreover, plasmatic NET by-products (e.g., MPO–DNA complexes) were found to be higher in extremely obese or diabetic patients (82, 123). The authors of these studies also speculated about the tangled association between NETs, chronic obesity, and diabetes, and how these could lead to long-term pathologies, such as delayed wound healing, increased cardiovascular disease, and thromboembolic events following COVID-19.

## NETosis and NET-based Therapeutic Approaches to COVID-19

With the growing recognition of the roles of NETosis and NETs in COVID-19, a few treatment approaches have recently been reported that inhibit NETosis and NET release, or promote NET degradation (**Table 1**). The first approach involves interfering with SARS-CoV-2 to stimulate neutrophils or antagonize the function of other extracellular pro-NETosis factors. For example, antigen–antibody complexes can stimulate FcγRIIA receptors on neutrophils to induce NETs in COVID-19 *via* spleen tyrosine kinase (SYK) phosphorylation and downstream signaling (124). R406 is the active ingredient of the SYK inhibitor, fostamatinib, which suppresses NETosis in neutrophils from healthy donors when activated by COVID-19 patient plasma (124). Furthermore, a synthetic glycopolymer agonist of sialic acid-binding immunoglobulin-type lectins (Siglec)-9, a checkpoint receptor in neutrophils, might reduce NETosis triggered by viral PAMPs (e.g., R848) or COVID-19 patient plasma (126). In COVID-19, the complement system is also overactive, and complement enriched NETs or the by-product of complement activation, known as anaphylatoxins, are linked to clinical symptoms of COVID-19 (32). Carboxypeptidase B2 (CPB2) is a natural plasma enzyme that degrades C3a and C5a anaphylatoxins (142). Zhang et al. found that recombinant CPB2 inhibits NET production in neutrophils isolated from COVID-19 patients and reduces the damage to cultured vascular endothelial cells (125). Other research has also revealed that antibodies against cytokines like IL-8, IL-6, and IL-1β not only reduce the resolution of inflammation but also block their pro-NETosis function (70, 133, 143). The second strategy targets the intracellular mechanisms that drive NETosis in response to SARS-CoV-2 infection. For example, vitamin C has been tested in phase 2 clinical trials aimed at reducing COVID-19-associated mortality by reducing excessive activation of the inflammatory response (135). Of note, vitamin C is an antioxidant that significantly attenuates PMA-induced NETosis in healthy neutrophils by scavenging ROS (144). Therefore, vitamin C may also inhibit NETosis and NET production in COVID-19. Proteases are important mediators in the NETosis process. Antiproteases that inhibit histone deacetylase (e.g., Belinostat), block NE activity (Alvelestat), or inhibit Gasdermin D (Disulfiram) have been shown to inhibit NETosis. These antiproteases have undergone preclinical (136) or clinical testing (11, 60) to demonstrate their efficacy in COVID-19. PAD4 is another crucial pro-NET enzyme that is upregulated in COVID-19 and is responsible for NET release (138, 145). Therefore, the use of PAD4 inhibitors, such as Cl-amidine, YW-56, and GSK484, may prevent both the prothrombotic and proinflammatory effects of SARS-CoV-2 by reducing NET production (138). For example, Cl-amidine has been shown to suppress NET release in both SARS-CoV-2-infected healthy neutrophils and blood neutrophils from COVID-19 patients (13). Cl-amidine also prevents apoptosis of lung epithelial cells due to NET release, suggesting that PAD4 inhibitors could be used to prevent immunothrombosis and lung injury in COVID-19 (13, 138). The third strategy involves mediating NET degradation. In fact, COVID-19 has been found to have a deficiency in NET clearance, resulting in pulmonary thrombo-inflammation (55). DNase-1 and DNase-1L3 are important catalyzers involved in the dissolution of NETs and the prevention of thromboembolic events caused by NETs (141). In this regard, DNases are currently being tested in clinical trials for the treatment of COVID-19 (55, 139). In another study using proteomic profiling, Fisher et al. showed that recombinant DNase treatment lowers NET production to promote recovery in COVID-19 patients (146). More research into the mechanisms underlying NET formation in COVID-19 may pave the way for other novel therapeutic approaches (e.g., extracellular histone neutralizers or NET inhibitors) (140).

**Table 1:** Anti-COVID-19 drugs in preclinical or clinical development *via* NETosis and NETs targeting mechanisms.

Category	Drug name	Mechanisms of action	Status of clinical trials	Reference
NETosis inhibitors	Fostamatinib	SYK inhibitor	NCT04579393, Phase 2	(124)
	Carboxypeptidase B	Degrades C3a and C5a	N.D.	(125)
	pS9L	Siglec-9 agonist	N.D.	(126)
	Avdoralimab	mAb against C5a	NCT04371367, Phase 2	(127, 128)
	Metformin	Immunomodulatory	NCT04604678, Phase 2 NCT04625985, Phase 2	(129–131)
	Anakinra	IL-1β inhibitor	NCT04412291, Phase 2 NCT04603742, Phase 2	(70, 132)
	Sarilumab	IL-6 receptor antagonist	NCT04661527, Phase 2	(133)
	Tocilizumab	mAb against IL-6	NCT04331808, Phase 2	(134)
	Vitamin C	ROS scavenger	NCT04264533, Phase 2	(8, 135)
	Belinostat and Panobinostat	Histone deacteylase inhibitor	N.D.	(136)
	Alvelestat	Antiprotease	NCT04539795, Phase 2	(137)
	Disulfiram	Gasdermin D inhibitor	NCT04485130, Phase 2 NCT04594343, Phase 2	(60)
	Cl-Amidine, GSK 484	PAD 4 inhibitor	N.D.	(138)
NET degraders	Dornase alfa	Degrades cfDNA	NCT04409925, Phase 1 NCT04445285, Phase 2	(55, 139)
	Long-acting DNase-1	Degrades cfDNA	N.D.	(140)
	DNase-I pMNSs	Degrades cfDNA	N.D.	(141)

SYK, Spleen tyrosine kinase; NCT, National clinical trial; C3a, Complement 3a; C5a, Complement 5a; N.D., No date; mAb, Monoclonal antibody; pS9L, Cis-binding Siglec-9 agonist; IL-1β, Interleukin 1 beta; IL-6, Interleukin 6; ROS, Reactive oxygen species; cfDNA, Circulating free DNA; Long-acting DNase-1, Long-acting nanoparticulate DNase-1; DNase-I pMNSs, DNase-I-coated melanin-like nanospheres.

## Conclusions

SARS-CoV-2 infection triggers NETosis and NET formation in both circulatory and infiltrating neutrophils, resulting in pulmonary injury, extensive inflammation, and the formation of a typical COVID-19 thrombus. The characteristic COVID-19 NET-induced thrombus contributes to microvascular obstruction and organ damage. However, new evidence reveals the role of NETs in pathological processes other than thrombosis, such as chronic aberrant immunity and long-term COVID-19. Given the ongoing COVID-19 pandemic, more people may develop both acute and chronic symptoms following SARS-CoV-2 infection. In this regard, urgent interventions are required to suppress the triggering factors involved in these processes or otherwise speeding up the degradation machinery, both of which may help to reduce NETosis and avoid the negative consequences of NET formation. In addition, precise management will be necessary to avoid disruption of protective neutrophil activities and NET formation. Furthermore, because DNase treatment may enhance the release of breakdown products from NETs (e.g., histones and proteases), it is important to avoid unwanted negative effects when developing novel anti-COVID-19 therapies that target NETs (96).

## Author Contributions

XL conceived the review article, reviewed the literature, and made substantial revisions before submission. YZ reviewed the literature, contributed to the writing of the manuscript, and prepared the table. XC reviewed the literature and contributed to the writing of the manuscript. All authors contributed to the article and approved the submitted version.

## Funding

This work was supported by grants from the National Natural Science Foundation (81902015 and 81801964).

## Conflict of Interest

The authors declare that the research was conducted in the absence of any commercial or financial relationships that could be construed as a potential conflict of interest.

## Publisher’s Note

All claims expressed in this article are solely those of the authors and do not necessarily represent those of their affiliated organizations, or those of the publisher, the editors and the reviewers. Any product that may be evaluated in this article, or claim that may be made by its manufacturer, is not guaranteed or endorsed by the publisher.
